# Results of the Cologne Corona surveillance (CoCoS) study – a prospective population-based cohort study: incidence data and potential underestimation of new SARS-CoV-2 adult infections by health authorities

**DOI:** 10.1186/s12889-022-13745-1

**Published:** 2022-07-19

**Authors:** Max Oberste, Lynn-Marie Pusch, Rebecca Roth, Kija Shah-Hosseini, Jana Schmitz, Eva Heger, Felix Dewald, Claudia Müller, Luise Stach von Goltzheim, Clara Lehmann, Michael Buess, Anna Wolff, Gerd Fätkenheuer, Gerhard Wiesmüller, Florian Klein, Kerstin Daniela Rosenberger, Florian Neuhann, Martin Hellmich

**Affiliations:** 1 Institute of Medical Statistics and Computational Biology, Medical Faculty and University Hospital Cologne, University of Cologne, Robert-Koch-Straße 10, 50931 Cologne, Germany; 2 Institute of Virology, Medical Faculty and University Hospital Cologne, University of Cologne, Fürst-Pückler-Straße 56, 50935 Cologne, Germany; 3 Department of Internal Medicine, Medical Faculty and University Hospital Cologne, University of Cologne, Kerpener Str. 62, 50931 Cologne, Germany; 4 Cologne Health Authority, Cologne, Germany; 5 Heidelberg Institute of Global Health, University Heidelberg, Heidelberg, Germany; 6 School of Medicine and Clinical Sciences, Levy Mwanawasa Medical University, Lusaka, Zambia

**Keywords:** Coronavirus, COVID-19, Pandemic, Surveillance, Incidence, Underestimation

## Abstract

**Background:**

Current incidence estimates of SARS-CoV-2 in Germany rely to a large extent on case notifications. However, the large number of mild or asymptomatic infections is likely to result in underestimation. Population-based studies can provide valid estimates of the SARS-CoV-2 incidence and thus support health authorities to monitor the epidemiological situation and to initiate, maintain, strengthen or relax effective countermeasures.

**Methods:**

This study was conducted in Cologne, Germany. Six-thousand randomly drawn Cologne residents, 18 years of age or older, were contacted by mail in March 2021. Study envelopes contained a kit for self-administered saliva sample and access details to a questionnaire on sociodemographic characteristics, previous positive SARS-CoV-2 RT-qPCR and completed COVID-19 vaccinations. Participants were again invited for a second round in June 2021, while those who declined participation were replaced by additional randomly drawn Cologne residents in order to reach a total of 6000 potential participants again. The saliva samples were sent to the laboratory by mail and tested for SARS-CoV-2 using RT-qPCR. The incidence estimates were adjusted for sensitivity and specificity of the test procedure and compared with the official numbers of new SARS-CoV-2 cases in the adult Cologne population.

**Results:**

The first surveillance round in March 2021 (response rate: 34.08%, *N* = 2045) showed a SARS-CoV-2 seven-day incidence of 85 cases per 100,000 adult Cologne residents (95% CI: 9 to 319). In the same period, the officially registered cases were 125 per 100,000. The second surveillance round in June 2021 (response rate: 36.53%, *N* = 2192) showed a seven-day incidence of 27 per 100,000 adult Cologne residents (95% CI: 1 to 142), while the official figures for newly registered SARS-CoV-2 cases in the same period were 15 per 100,000.

**Conclusions:**

The incidence estimates do not indicate relevant underestimation of new SARS-CoV-2 infections based on case notification. Regular use of the surveillance method developed here may nevertheless complement the efforts of the health authorities to assess the epidemiological situation.

**Trial registration:**

DRKS.de, *German Clinical Trials Register* (DRKS), Identifier: DRKS00024046, Registered on 25 February 2021.

**Supplementary Information:**

The online version contains supplementary material available at 10.1186/s12889-022-13745-1.

## Background

After its first appearance in December 2019 in Wuhan, China, the novel coronavirus SARS-CoV-2 (Severe Acute Respiratory Syndrome-Coronavirus-2) spread across the globe in just a few weeks. In Germany, as in many other countries, the government introduced drastic countermeasures at an early stage of the pandemic in order to avoid overburdening the health system and especially the intensive care units. German politics and public health authorities are assessing the current course of the pandemic on the basis of various epidemiological indices and indicators. They are important aids when making decisions about maintaining, tightening or releasing countermeasures that have been initiated. The number of newly confirmed SARS-CoV-2 infections within a specified period of time plays a crucial role among the key epidemiological indicators of the pandemic. It is usually reported as the accumulated number of positive SARS-CoV-2 ‘Real Time quantitative Polymerase Chain Reaction tests’ (RT-qPCR) over the past seven days and given per 100,000 inhabitants of the general population [1, 2]. However, the accumulated number of positive SARS-CoV-2 RT-qPCR within the last seven days per 100,000 inhabitants might be rather a rough approximation of the true incidence of SARS-CoV-2. There is a risk that the official bodies underestimate the true extent of the spread of SARS-CoV-2 in the population on the basis of the accumulated positive SARS-CoV-2 RT-qPCR within the last seven days per 100,000 inhabitants [3–5].

The here presented ‘Cologne Corona Surveillance (CoCoS) Study’ was carried out to examine the risk of possible underestimation of the true incidence of SARS-CoV-2 by the official figures. It used an active cluster surveillance strategy in the city of Cologne, Germany, at two time-points of the pandemic (March 2021 and June 2021). The study was planned and carried out in close cooperation with the Cologne health authorities.

## Methods

A detailed description of the study setting, design, sample and procedure has already been published as study protocol [6]. In summary, we combined a cross-sectional and longitudinal design. A random sample of 6000 Cologne citizens who were 18 years of age or older, was drawn from the municipal-registration office. The study consisted of two surveillance rounds (March 16 to March 29, 2021 and June 5 to June 18, 2021). All potential participants drawn in the sample received a participation package by post on March 16 and June 5, 2021. In addition to the letter of invitation to take part in the study, the package contained a saliva sample kit and a pre-addressed, stamped UN3373 envelope. The saliva sample kit consisted of the saliva collection system, Salivette® [7]. This system was recently validated as a method for self-collection of saliva samples in order to test for SARS-CoV-2 [8]. To take part in the study, participants had to return their saliva sample during the 14 days of each surveillance round. For the second surveillance round, participants who declined informed consent in the first surveillance round were replaced in order to again reach 6000 potential participants. The newly invited participants were drawn from a second sampling from the municipal-registration office.

Data on non-participation was collected. Basic sociodemographic characteristics of the non-participants can be described, as these data were recorded as part of the sampling from the municipal-registration office. In addition, non-participants were asked to provide reasons for not participating in the study. The invitation letter provided for the following options to be ticked: ‘I do not consider the topic to be important’/‘I do not want to spend any time on the topic / I do not want to take the saliva sample’/‘I have concerns about data protection’. Non-participants could also indicate other reasons in a free text field.

### Laboratory analysis

The participants’ saliva samples were analysed at the University of Cologne, Faculty of Medicine and University Hospital Cologne, Institute of Virology. All laboratory methods have also been described in detail in the study protocol that has already been published [6]. In brief, we determined SARS-CoV-2 using RT-qPCR in a pool of each 10 samples, containing one ml of pooled saliva. For SARS-CoV-2 detection, either the COBAS 6800 (Roche Diagnostics) or Alinity m (Abbott) instruments equipped with their respective SARS-CoV-2 detection kits were used. One ml of each sample of a positive pool were re-tested individually as described above. In a recently published meta-analysis, the sensitivity of RT-qPCR from saliva samples for the detection of SARS-CoV-2 was quantified as 83.2% (95% CI 74.7 - 91.4%) compared with the gold standard (RT-qPCR from nasopharyngeal swab) [9]. The specificity of the test methods used here is close to 100% [10]. Given the low prevalence of SARS-CoV-2 in the population, specificity is of central importance with regard to the accuracy of prevalence and incidence estimates [11]. In the present study, three positives were identified from a total of 4237 tests. Even if all of these had been false positive, this would still correspond to a specificity of the test method of 99.93% (95% CI 99.79–99.99%).

For analysis of the variants of SARS-CoV-2 positive samples, 500 μl of saliva were used to purify nucleic acids with the MagNa Pure 96 automatic nucleic acid extraction instrument and the Viral NA large volume kit. One-hundred μl were used for elution. Of the extracted RNA, 5 μl were used in a qPCR using the VirSNip SARS-CoV-2 Spike A23063T N501Y, del21765–770 del HV69/70 and G23012A E484K assay according to the manufacturer’s instructions (TIBMolBiol, Berlin, Germany). Melting analyses were performed in a LightCycler® 480 II (Roche Diagnostics).

Positive SARS-CoV-2 PCR tests are notifiable in Germany. Accordingly, the participants who tested positive were reported to the authorities, who immediately informed them about their infection and initiated appropriate quarantine measures. The participants were informed in detail about this procedure as part of the consent to participate.

### Questionnaire

Complementing the saliva sample, the participants were asked to share study-related data in a questionnaire during both surveillance rounds of the study. The participants could fill out the questionnaire independently online or with the help of the study team via telephone interview. A detailed description of the questionnaire can be found in the previously published study protocol [6]. The analytical focus in this article includes the sociodemographic items of the questionnaire, such as gender, age, school education, employment status, primary citizenship, number of household members, as well as items on statements of previous positive SARS-CoV-2 PCR-tests and possibly completed Corona Virus Disease 19 (COVID-19) vaccinations.

### Statistical analysis

Statistical analysis was performed using SPSS (IBM Corp., Version 26.0, Armonk, NY, USA) and the statistical software R (R Foundation for Statistical Computing, Vienna, Austria). The absolute and relative frequencies of basic sociodemographic characteristics of the two samples of 6000 potential participants each were determined using the sampling data from the municipal-registration office. The absolute and relative frequencies of the more in-depth sociodemographic information from the questionnaire were determined for each of the two participant samples. The sociodemographic information of the potential participants and the participant samples were compared with the official statistics of the city of Cologne on Cologne’s adult general population. These data are open source [12].

From the information provided by the participants on previous positive PCR-tests for SARS-CoV-2, the proportion of participants who had gone through an infection was estimated for each of the two surveillance rounds. Weighting was given for the Cologne districts. Districts that were below the detection limit were estimated with 0.499 positive cases. We found this statistical measure to be necessary to improve the accuracy of our study data, as statistically the true incidence in those districts was slightly under our detection level, rather than zero. The estimates of the cumulative cases were compared with the official data up to the day before the beginning of the respective surveillance round (March 15 and June 4, 2021). These data were made available by the health authorities of the City of Cologne upon our request. The requested data are provided in the Supplementary Material to this article. The absolute and relative frequencies of the information provided by the participants on COVID-19 vaccinations were calculated for each sample of the two rounds of surveillance. These were compared with the official statistics of the Robert-Koch-Institute on Cologne’s adult general population up to the beginning of the respective surveillance round (up to March 15/June 4, 2021). These data are open source [13].

The prevalence of SARS-CoV-2 infections was estimated as the quotient of the positives and the total sample of participants in each surveillance round and adjusted for the sensitivity and specificity of the test method using a Bayesian approach [14]. From the prevalence, the incidence was estimated as the rate of new cases per day by dividing by the average duration of illness of a SARS-CoV-2 infection [15]. In their review, Walsh and colleagues [16] recently state that the average duration of illness for mild to moderate disease courses is around 10 days. From the estimate of the new cases rate per day, a 7-day incidence per 100,000 participants was calculated by multiplying the incidence estimate by 7 days and 100,000 inhabitants. These figures were compared with the official information that the health authorities of the City of Cologne made available to us on request (these data are also provided in the Supplementary Material to this article). The mean values of the daily new infections officially registered in the adult Cologne general population during the 14-day study periods (March 16 to 29 and June 5 to 18, 2021) were used as a basis for the comparison with the incidence estimates obtained from the study.

## Results

### Study recruitment

**Fig. 1:**
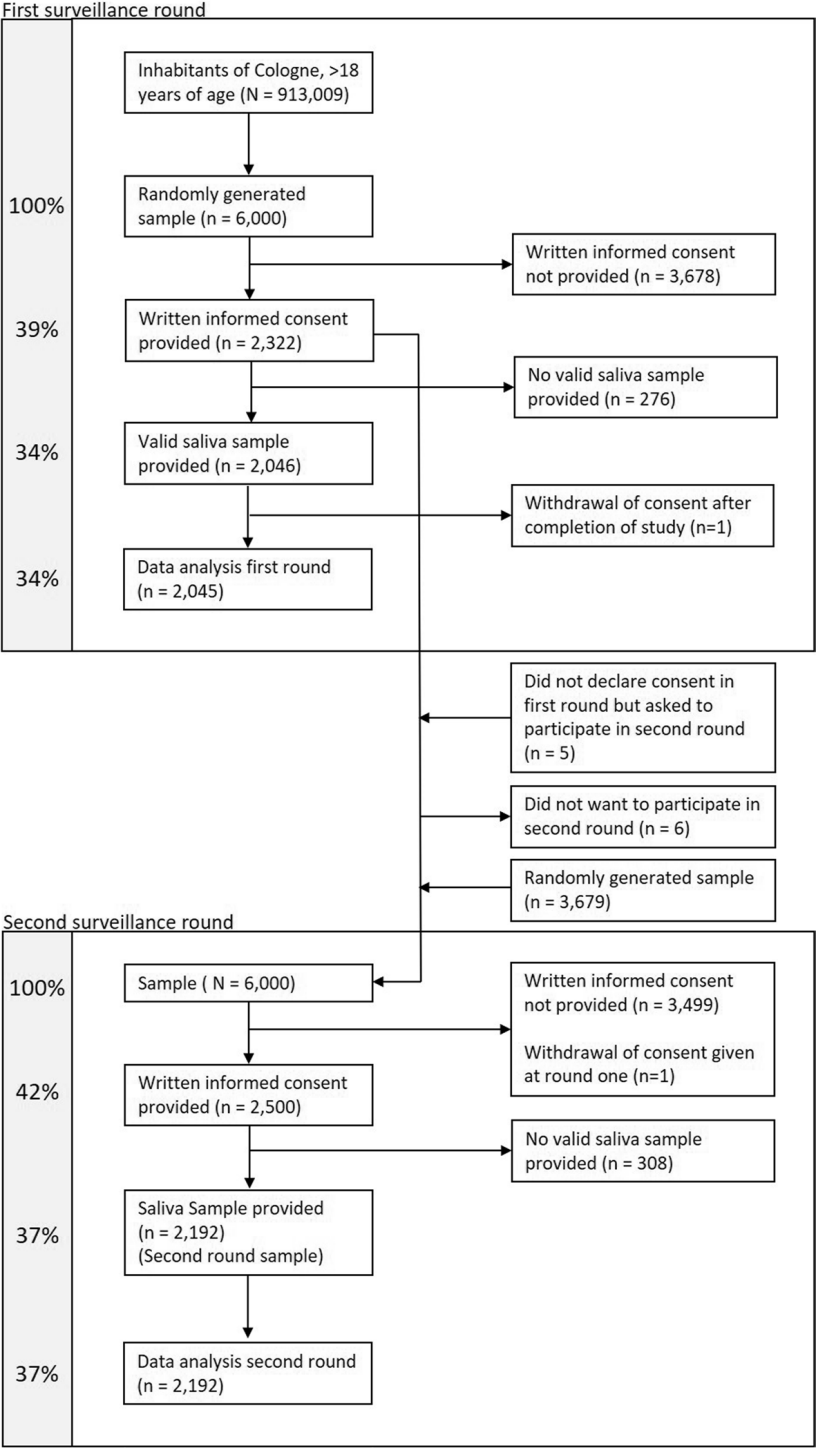
Flow chart of enrolment and testing at each surveillance round

The study flow chart is shown in Fig. 1. The reasons why 276 (11.89%) of the 2322 participants who gave their consent to the first surveillance round of the CoCoS study, did not submit a valid saliva sample were incorrect handling of the Salivette® saliva sample system, forgetting to pack the sample in the return envelope and sending the sample back only after the end of the surveillance round. Two-hundred-seven of the 3678 potential participants who decided not to part in the first surveillance round, made use of the opportunity to give reasons for not taking part (for most common reasons see Table 1).

**Table 1:** Most common reasons for non-participation stated in the tick boxes and the free text field of the invitation letter

	**First surveillance round:**		
	**Reasons**	**Number of Counts**	**Percentage of 207 stated reasons**
Tick Answers	Did not want to undergo saliva sampling	42	20.29%
	Did not want to spend as much time on the topic	26	12.56%
	Data privacy concerns	25	12.08%
	Did not think the topic was important	6	2.90%
Free text field answers	Medical and health issues (not associated with COVID-19)	21	10.14%
	High age	13	6.28%
	Longer stay outside Cologne	10	4.83%
	Taking regular tests for SARS-CoV-2 infection	9	4.35%
	No access to internet / No technical equipment	8	3.86%
	Too much effort	8	3.86%
	General criticism of the study	8	3.86%
	Already vaccinated	6	2.90%
	**Second surveillance round:**		
	**Reasons**	**Number of Counts**	**Percentage of 96 stated reasons**
Tick Answers	Did not want to undergo saliva sampling	15	15.63%
	Did not want to spend as much time on the topic	12	12.50%
	Data privacy concerns	7	7.29
	Did not think the topic was important	3	3.13%
Free text field answers	Medical and health issues (not associated with COVID-19)	12	12.50%
	Already vaccinated	10	10.42%
	Longer stay outside Cologne	8	8.33%
	Personal reasons	4	4.17%
	Missed deadline for participation	4	4.17%
	High age	3	3.13%
	No access to internet/No technical equipment	3	3.13%
	No acceptance of gender-sensitive language	3	3.13%

The participants from the first surveillance round were also invited to the second surveillance round in June 2021. The total number of re-contacted persons from the first surveillance round for participation in the second round was 2321. They correspond to 38.68% of the 6000 potential participants contacted for the second surveillance round. In order to fill the number of potential participants for the second surveillance round to 6000 Cologne citizens, a further 3679 (61.32%) were randomly drawn from the general adult population of Cologne.

Of the 2321 people from the first round who were also invited to the second round, 1499 (64.60%) gave their consent to participate in the second surveillance round of the study. Of the people who were newly invited to the second round, 27.21% gave their consent to participate. The reasons why 308 (12.32%) of 2500 people who gave their consent to participation in the second surveillance round, did not provide a valid saliva sample were incorrect handling of the Salivette® saliva sample system, forgetting to pack the sample in the return envelope and sending the sample back only after the end of the examination round.

Ninety-six of the 3499 potential participants in the second surveillance round who decided not to participate in the study gave reasons for not participating. (for most common reasons see Table 1).

It is worth mentioning that not only the new participants, but also those who had already participated in the first round, received detailed instructions on how to collect saliva with the Salivette® system. In this way, failures due to errors in the collection of saliva should be further reduced. Of the 276 people who had agreed to participate in the first round but had not given a valid saliva sample, 140 people (50.72%) took part in the second round. Of these 140 people, 101 people (72.14%) submitted a valid saliva sample for the second round.

### Description of the study participants

Sociodemographic characteristics and SARS-CoV-2 specific information of the 6000 randomly drawn citizens of Cologne invited to participate in each of the two surveillance rounds (potential participants), of the actual participants in both surveillance rounds, as well as of the adult general population of Cologne is summarized in Table 2.

**Table 2:** Sociodemographic characteristics and SARS-CoV-2 specific information of the CoCoS study participants (first and second round) compared to the potential participants and to the general adult Cologne population

	General adult Cologne population		Potential participants CoCoS round #1		Participants CoCoS round #1		Potential participants round #2		Second examination CoCoS study	
**Sample**										
Participants (18 yrs. or older)^1^	913,009	100%	6000	100%	2045	34.08%	6000	100%	2192	36.53%
**Gender**^**a**^										
Female	470,577	51.54%	3090	51.51%	1088	53.20%	3086	51.44%	1189	54.24%
**Age**^**a**^										
18–34 years (%)	270,538	29,63%	1795	29.92%	518	25.33%	1662	27.70%	506	23.08%
35–59 years old (%)	388,678	42,57%	2560	42.67%	951	46.50%	2640	44.01%	996	45.44%
60–74 years old (%)	153,890	16,86%	1007	16.79%	418	20.44%	1095	18.25%	474	21.62%
75 years or older (%)	99,903	10,94%	637	10.62%	158	7.73%	602	10.04%	216	9.85%
**No. of household members**										
Average	913,009	1.88	NA	NA	1580	2.36	NA	NA	1689	2.32
1–2	438,859	77.68%	NA	NA	1055	66.77%	NA	NA	1158	68.56%
3–4	106,314	18.82%	NA	NA	455	28.80%	NA	NA	472	27.95%
5 or more	19,800	3.50%	NA	NA	70	4.43%	NA	NA	59	3.49%
Missing values^b^	–	–	–	–	465	22.74%	–	–	503	22.95%
**School education**										
No school leaving certificate	36,520	4.00%	NA	NA	8	0.50%	NA	NA	8	0.46%
Secondary school diploma	374,333	41.00%	NA	NA	400	24.99%	NA	NA	419	24.22%
High school graduation	502,155	55.00%	NA	NA	1193	74.52%	NA	NA	1303	75.32%
Missing values^b^	–	–	–	–	444	21.71%	–	–	462	21.08%
**Employment status**										
Student/apprenticeship	122,849	13.46%	NA	NA	163	9.85%	NA	NA	161	9.10%
Employed	582,613	63.81%	NA	NA	971	58.71%	NA	NA	1025	57.91%
Self-employed	NA	NA	NA	NA	179	10.82%	NA	NA	201	11.36%
Retired	NA	NA	NA	NA	243	14.69%	NA	NA	288	16.27%
Unemployed	45,225	4.60%	NA	NA	38	2.23%	NA	NA	40	2.26%
Other^2^	NA	NA	NA	NA	60	3.63%	NA	NA	60	3.40%
Missing Values^b^	–	–	–	–	391	19.12%	–	–	422	19.25%
**Primary citizenship**^**a**^										
German	727,503	79.68%	4677	77.96%	1894	92.62%	5039	84.00%	2067	94.30%
other than German	185,506	20.32%	1322	22.04%	151	7.38%	960	16.00%	125	5.70%
**Cumulative SARS-CoV-2 cases**										
Start of the pandemic until March 14, 2021	32,119	3.52%	NA	NA	67	3.71% [2.78, 4.64%]	NA	NA	NA	NA
Start of the pandemic until June 6, 2021	46,195	5.06%	NA	NA	NA	NA	NA	NA	90	4.61% [3.60, 5.61%]
**Vaccination rate until March 15, 2021**										
Vaccinated at least once	82,718	9,06%	NA	NA	159	9.66%	NA	NA	NA	NA
Vaccinated twice	39,684	4.35%	NA	NA	59	3.58%	NA	NA	NA	NA
Not vaccinated	839,291	90.94%	NA	NA	1487	90.34%	NA	NA	NA	NA
Missing Values^b^	NA	NA	NA	NA	399	19.51%	NA	NA	NA	NA
**Vaccination rate until June 4, 2021**										
Vaccinated at least once	529,248	57,97%	NA	NA	NA	NA	NA	NA	1396	79.82%
Vaccinated twice	220,987	24.20%	NA	NA	NA	NA	NA	NA	589	33.68%
Not vaccinated	383,761	42.03%	NA	NA	NA	NA	NA	NA	353	20.18%
Missing Values^b^	NA	NA	NA	NA	NA	NA	NA	NA	443	20.21%

^a^Information obtained directly from the population register

^b^While the percentages on the variables expressed relate to the respondents of the respective questionnaire item, the percentages on the missing values relate to the sample (*N* = 2045/*N* = 2192)

In both surveillance rounds, the actual participants of the CoCoS study were reasonably representative of the general adult population with regard to gender, age, size of household, employment status, and past SARS-CoV-2 infections. However, the actual participants in both surveillance rounds of the CoCoS study had a higher level of schooling than the general adult population in Cologne (74.52%/75.32% versus 55.00% with High school graduation). Moreover, people from Cologne with primary citizenship other than German were underrepresented among the actual participants in both surveillance rounds of the CoCoS study (7.38%/5.70% versus 20.32% with primary citizenship other than German). In the second surveillance round, the proportion of actual participants who had already been vaccinated were higher than in the adult general population of Cologne (vaccinated once: 79.82% versus 49.00%, vaccinated twice: 33.68% versus 21.22%) (for official figures concerning vaccination rates in Cologne see [13]).

### Prevalence and incidence of SARS-CoV-2

In the first surveillance round of the CoCoS study, three of the 2045 participants tested positive for SARS-CoV-2 (viral loads (measured in E-gene Ct values): 28, 29, and 36, SARS-CoV-2 variants: two times Alpha variant, one could not be determined because of a low viral load). None of the participants who tested positive in this study had previously been vaccinated against the SARS-CoV-2 virus. Two of the three infected individuals were already known to the health authorities. The infected person unknown to the health authorities was informed immediately and reported to the health authorities.

For the first surveillance round of the CoCoS study, the estimated prevalence of SARS-CoV-2 infections corrected for the sensitivity and specificity of the laboratory tests was 0.122% of the adult Cologne population, with a 95% confidence interval ranging from 0.013 to 0.457%. From this, based on a disease duration estimate of ten days [16], the incidence of new SARS-CoV-2 infections per day was estimated to be 0.0122% with a 95% confidence interval that ranges from 0.0013 to 0.0457%. The conversion to a 7-day incidence resulted in 85 per 100,000 inhabitants with a 95% confidence interval ranging from 9 to 319 per 100,000 inhabitants. From March 16 to March 29, 2021, the health authorities reported an average case notification rate of 125 per 7 days per 100,000 inhabitants.

Corrected for the sensitivity and specificity of the laboratory test, a prevalence estimate of 0.038% was calculated for the second surveillance round of the study with a 95% confidence interval ranging from 0.001 to 0.0202%. The resulting incidence estimate is accordingly 0.0038% with a 95% confidence interval of 0.0001 to 0.0202%. The conversion to a 7-day incidence estimate resulted in 27 per 100,000 inhabitants with a 95% confidence interval that ranges from 1 to 142.From June 5 to 18, 2021, the health authorities reported an average case notification rate of 0.0021% or a 7-day case notification rate of 15 per 100,000 inhabitants.

## Discussion

The here presented CoCoS study provides two SARS-CoV-2 incidence estimates for two very different phases of the pandemic (high incidence phase in March 2021 and low incidence phase in June 2021) obtained from randomly drawn population samples. The estimates of the incidence of new SARS-CoV-2 infections per day derived from the results of the study were close to the officially registered cases. In the first round of surveillance, the 7-day incidence estimate was even slightly below the 7-day incidence based on the officially registered cases, at 85 versus 125 cases per 100,000 inhabitants. In the second surveillance round, the 7-day incidence estimate was slightly higher than the 7-day incidence based on the officially registered cases, at 27 versus 15 cases per 100,000 inhabitants. These findings do not indicate underestimation of the true incidence on the basis of the officially registered cases, as has been shown in other studies [17, 18].

The confidence intervals for the incidence estimates are very wide reflecting low precision of incidence estimates. To narrow down confidence intervals in future surveillance rounds sample size could be increased. This could be achieved with increasing the response rate. The response rates in each round (38.68 and 41.67%, respectively) are comparable to the response rates from similar studies [19, 20]. However, alternative and easier to handle methods for taking saliva samples, such as the ‘lollipop method’ [21], are currently evaluated for self-administered use [22] and could increase the response rates in future surveillance rounds.

A striking observation is that the response rate was significantly lower among first time invitees of the second round compared to the first round (27.21% versus 38.68%). Since the procedure for recruiting new potential participants was identical in the first and second round, external factors seem likely to be the explanation. During the period of the first investigation, comprehensive and free of charge SARS-CoV-2 tests were not yet accessible for all citizens. The free PCR testing in the study may therefore have been an incentive to participate. This assumption is supported by the feedback from the non-participants in the first round who explain their non-participation with the possibility of regular tests apart from the study. When the second surveillance round was carried out, test centres were introduced everywhere in Cologne, where every citizen could be tested easily and free of charge. In addition, a large part of the Cologne population was already vaccinated in the second round, which, according to the feedback of non-participants, was often cited as a reason for not taking part in the second round.

If the incentive of cost-free testing no longer works due to the citywide development of test centres and the increasing spread of COVID-19 vaccinations, other incentives could stabilize and expand the response rate in future surveillance rounds. While monetary incentives are effective but often not affordable and difficult to implement in the context of population-based studies, the response rate can be increased significantly with simple non-material incentives [23]. An effective non-material incentive is the feeling of participating in a meaningful study [24]. The invitation letter for the CoCoS study contained extensive information on the study procedure, the voluntary nature of participation, contact persons for queries and data protection, as well as instructions for taking the saliva sample. Possibly more information about the importance of the study as part of effective countermeasures to contain the pandemic would have increased the response rate even further. In future rounds, the relevance of the study should be stressed in the invitation letter.

Reminders to participate are commonly seen as the most important factor in increasing the response rate of postal surveys [25]. Future surveillance rounds could send postcards or other means like text-messages (if phone-number or e-mail address is available) to the potential participants during the second half of the 14-day surveillance period to remind them of the study and ask them to participate. This is all the more important that especially those potential participants who would otherwise remain under-represented are motivated to participate through reminder campaigns [26].

The CoCoS study combined cross-sectional and longitudinal design. The same approach has been taken, for example, by the Robert-Koch-Institute (RKI), the German national public health institute [27]. Participants of previous waves are invited to continue, and drop-out is filled-up by new, randomly selected persons. This approach proved to be successful in terms of the response rate. While only 27.21% of those newly invited to the second surveillance round took part, 64.60% of those who were re-invited also took part in the second round. The approach not only to invite the people with consent to participate and a valid saliva sample to the second round, but all people who declared their participation in the first round (including those with consent but missed to send a valid saliva sample), proved to be successful. Most of the participants who did not submit a valid saliva sample for the first round took part in the second round and then managed to submit a valid saliva sample. This is presumably due to the detailed instructions on how to collect the saliva sample that were given not only to the newly invited potential participants, but also to the re-invited potential participants in the second round. In future rounds, however, only those participants who did not submit a valid saliva sample in the previous round could receive detailed instructions on how to take saliva samples again and not all re-invited participants in general. In this way, printing and paper costs could be saved and the reading effort for the other re-invited participants could be reduced.

The rolling cohort design also has disadvantages. Selection effects can increase from round to round and threat the representativeness of the study sample. The validity of the incidence estimates depends on the representativeness of the sample. Such a trend cannot be seen in the data from the first to the second surveillance round. However, of the 6,000 randomly drawn citizens of Cologne who were invited to take part in the study and who represented potential participants, only their name, address, age, and gender were available. These data could be obtained from the population register. Since only some of these potential participants eventually took part in the study, no further data on the potential participants could be obtained. This applies to the size of the household, school education, employment status and primary citizenship, as well as past positive PCR tests or previous COVID-19 vaccinations. The lack of this data does not allow any reliable conclusions to be drawn about possible selection effects. If the monitoring system developed as part of the CoCoS study is to be permanently established, a sentinel cohort should, however, be established that is representative of the population and that is regularly examined. As an incentive to be included in this sentinel cohort, the great importance could be placed in the foreground non-materially and / or material monetary incentives could be pointed out (e.g., regular health check-ups, information of the current course of the pandemic).

A critical examination of the representativeness of the participant samples in the two surveillance rounds shows that the participants differed from the general adult population in Cologne, especially in terms of level of school education and primary citizenship. Correlations between the level of education and the probability of a SARS-CoV-2 infection have already been reported for the Cologne area. It was also shown that SARS-CoV-2 infections are more common in Cologne districts with a high proportion of people with primary citizenship other than German [28]. Therefore, the under-representation of persons with lower levels of school education and of non-German citizens of Cologne could indicate an underestimation of the incidence in our study. One reason for the under-representation of people with lower school education in the participant sample could have been the length of the invitation letter. The letter of invitation is eight pages in its current form. The amount of text could put off less educated potential participants. A shorter and more concise version, perhaps with easy language, could motivate Cologne citizens with less school education to participate. In order to attract more people from Cologne with primary citizenship other than German to participate in future rounds, a reference to a corresponding website in the mother tongues of the largest groups of foreigners in Cologne could be included in the invitation letter. To support a low threshold access to studies like CoCoS, multilingual options or cooperation with the city’s office for integration could be considered.

The vaccination quota of the participants in the first round was similar to the officially reported vaccination quota for Cologne. In the second round, however, the participants had a markedly higher vaccination rate than officially reported [13]. The significantly higher vaccination rate in the participant sample of the second surveillance round, compared with the official figures, could represent an actual difference to the normal population. This would be problematic as it can lead to biases in the incidence estimates. Vaccinated people can still carry the SARS-CoV-2 virus. However, they become ill less often, for a shorter period of time and less severely than those who have not been vaccinated [29]. Furthermore, the Robert Koch Institute recently published a report on this with its own surveys in which it assumes 10% and more vaccinations are not reported [30]. Another risk of bias caused by the higher proportion of vaccinated people in the study sample is difference in behaviour and attitudes towards non-pharmacological measures to prevent infections and exposure to SARS-CoV-2 that are associated with the COVID-19 vaccination status. Some evidence suggests that those willing to be vaccinated also show greater compliance to non-pharmacological measures [31]. However, other findings suggest that completed COVID-19 vaccinations do not change behaviours that prevent infection or tend to decrease somewhat [32].

One limitation of the study is that it only includes people over the age of 18. Children and adolescents play an important role in the spread of SARS-CoV-2, especially due to the lack of approved vaccines or vaccinations that have not yet achieved full coverage [33, 34]. However, children and adolescents represent a special clientele who could hardly be reached with a mail-based surveillance strategy conceived here. As part of the nationwide research network “Applied Surveillance and Testing” (B-FAST), research on SARS-CoV-2 surveillance for under and over 18-year olds were subdivided. The procedure of the surveillance strategy developed for children and adolescents was based on testing in kindergartens and schools and first results have been published and can be read there [21]. In future surveillance rounds, the here applied mail-based surveillance strategy for adults and the surveillance strategy based on visits in kindergartens and schools should be combined to get a clear picture of the COVID-19 situation.

Another limitation of the CoCoS study are the periods of the two surveillance rounds of 14 days each. Since the incidence of SARS-CoV-2 can fluctuate considerably within 14 days and the probabilities of detecting acute SARS-CoV-2 infections change, this constitutes another limitation. The surveillance strategy developed here, however, assumes a fixed incidence or simplifies the actual procedure in which all samples are taken on the same day. This brings additional bias in the estimate of the incidence. The establishment of a sentinel surveillance cohort, who know the processes well, could enable the investigation period to be further restricted to up to 3 days. This would significantly increase the accuracy of the estimate and the comparison with the official figures.

Further, without blood drawing in surveys like ours, SARS-CoV-2 antibodies cannot be determined, hence it remains unclear how many participants already gone through a SARS-CoV-2 infection. Therefore, no statement can be made about possible underestimation of cumulative cases.

## Conclusion

The official numbers from the health authorities were within the 95% confidence interval of the incidence estimates determined in the study in both surveillance rounds. Nevertheless, regular application of the surveillance method developed here could complement the efforts of health authorities to assess the epidemiological situation. Regular surveillance or monitoring of population-based samples may give useful indicators both of distribution and determinants of SARS-CoV-2 infections. On the one hand, the official registration of infection is to a large extent based on symptomatic cases only, thus giving rise to potential underreporting, the detection of which was key objectives of our study. On the other hand, non-response in random population-based samples can bias the results in both directions. Thus, we suggest that both designs shall be used complementary to each other. Our data complement statistics from health authorities to support the local implementation or adaptation of countermeasures in order to control or contain the spread of SARS-CoV-2 infection. To narrow down confidence intervals a larger number of people invited into the study could be effective. Moreover, incentives, reminders to participate, and an easier to handle saliva sampling method should be used to increase the response rate.

## Supplementary Information

**Additional file 1.**

## Data Availability

The datasets used and/or analysed during the current study are available from the corresponding author on reasonable request.

## Abbreviations

DRKS: German Clinical Trials Register
SARS-CoV-2: Severe Acute Respiratory Syndrome-Coronavirus-2
RT-qPCR: Real time quantitative Polymerase Chain Reaction
CoCoS-study: Cologne Corona Surveillance Study
COVID-19: Corona Virus Disease 19
RNA: Ribonucleic Acid
